# Orthopedic Considerations with Eosinophilic Fasciitis: A Case Report and Literature Review

**DOI:** 10.1155/2012/865360

**Published:** 2012-06-28

**Authors:** Jason Samona

**Affiliations:** Michigan State University College of Osteopathic Medicine, 6304 Timberwood South, West Bloomfield, East Lansing, MI 48322, USA

## Abstract

Eosinophilic fasciitis (EF) or Shulman's disease is a very rare condition first described in 1974 by Dr. Shulman in patients with diffuse fasciitis and eosinophilia. Fewer than 300 cases have been reported worldwide in the past 35 years. The current understanding of the disease in the medical community relies only on a few large case series and multiple case reports. The proposed etiology, pathological mechanisms, and consensus for therapy are obscure or lacking. The presentation of the disease is variable, but certain signs and symptoms have been associated with EF. The extreme rarity of the disease, the large constellation of signs and symptoms, as well as the lack of knowledge about eosinophilic fasciitis and make this disease difficult to recognize and treat. Through the review of the literature, there is only one other case by Yamanishi where recurrent asthma has been seen to be associated with eosinophilic fasciitis. To the knowledge of the authors of this paper this patient represents the second recorded incident. The case described by the authors of this paper demonstrated an initial biopsy of mixed cell fasciitis including eosinophils, compared to the eosinophil-rich sample taken at a later date. This could be a unique aspect to the pathology of the disease not previously discovered. Similar scenarios were not noted in a review of the literature. A change in the pathological findings as shown in this case from non-eosinophil-rich sample to one rich in eosinophils is unique in a patient actively suffering from EF. The authors of this paper propose that an allergic reaction (at the patient's puncture site) occurred, which initially caused the left hand symptoms that led to the patient's first presentation to the hospital. This is a unique causative agent, not found in the review of the literature. Through a review of the literature and the presentation of this patient, the authors propose an underlying dysregulation of the immune system, leading to the initiation or synergistic perpetuation of EF. This is a unique outlook on the disease pathology, not explained much in the medical literature.

## 1. Introduction

Eosinophilic fasciitis (EF) or Shulman's disease is a very rare condition first described in 1974 by Dr. Shulman in patients with diffuse fasciitis and eosinophilia. Fewer than 300 cases have been reported world-wide in the past 35 years. The clinical spectrum of the disease has evolved since its initial description to reveal varying clinical presentations. The current understanding of the disease in the medical community relies only on a few large case series and multiple case reports [1–3]. The proposed etiology, pathological mechanisms, and consensus for therapy are obscure or lacking [4]. Therefore, the understanding of key aspects of the disease continues to evolve as time passes and more people are recognized as having this unique condition. Therefore, in combination with the rarity of the condition, the varying clinical presentations, and the scarcity of knowledge on the subject, clinical recognition of the disease and proper treatment are difficult to achieve [1–3].

There exists no clear consensus regarding the demographics of eosinophilic fasciitis. Clinical research has revealed a patient population of 1–88 years of age. Although, most reports consistently claim a mean age of onset between 40 and 50 years of age. Race and family history are not clear risk factors in developing the condition [2, 4], but it has been found to effect women and whites more often [2, 4, 5].

The etiology of eosinophilic fasciitis is under much debate. Among the proposed mechanisms are extreme physical exertion, ingestion of certain pharmaceuticals containing l-tryptophan, statin drugs, and various other agents [6, 7]. Exercise-induced eosinophilic fasciitis has been implicated in causing eosinophilic fasciitis in nearly 50% of cases in some studies. Other potential triggers include exposure to toxins, environmental, trauma, arthropod bites, and borreliosis [2]. In the review of literature, only one case report was found to claim association between relapsing eosinophilic fasciitis and bronchial asthma [8].

The presentation of the disease is variable, but certain signs and symptoms have been associated with EF. Among these presentations are bone pain or tenderness, carpal tunnel syndrome, muscle weakness, tenderness and swelling of the arms and legs (including joints), joint contractures, sclerodactyly, pitting edema, skin induration (in nearly 100% of patients), and thickened puckered skin (“peau d' orange” appearance) [1, 2, 4, 5]. In a landmark study of 52 cases observed at the Mayo Clinic, the disease process was found to affect virtually any body surface areas. Localized morphea was observed in 29% of the patients, arthritis in 40%, 56% had flexion contractures, and 23% presented with carpal tunnel syndrome. Nonspecific EMG changes were also seen in some patients. Associated hematologic diseases were found in nearly 10% of the patient population, which included thrombocytopenia, myeloproliferative disorder, myelomonocytic leukemia, and chronic lymphocytic leukemia. Peripheral blood eosinophilia was noted in 63% of the patients in this study, as compared to hypergammaglobulinemia in 35%, and elevated sedimentation rate in 29%. No matter what the cause, aberrant immune responses play a role in the pathology of the disease [5]. Hypergammaglobulinemia, antinuclear antibodies, and rheumatoid factor have all been shown to be associated with this disease at times [5–7, 9]. One study described the development of aplastic anemia and Hashimoto's thyroiditis in patients [4]. The signs and symptoms initially present suddenly, with the onset of painful, tender, edematous, and erythematous extremities. The disorder typically progresses fairly rapidly, within weeks to months [10]. Due to conflict surrounding proposed causative agents, the introduction of the triggering event and the onset of symptoms do not have a clear time correlation.

Tests to aid in the diagnosis of the disease include evaluation of gamma globulins (hypergammaglobulinemia), erythrocyte sedimentation rate (ESR), magnetic resonance imaging, and muscle and skin biopsies. Definitive diagnosis of the disease is made by a full thickness skin to muscle biopsy showing inflammation and thickening of collagen bundles in the superficial muscle fascia, with infiltration of lymphocytes and plasma cells. Eosinophils are at times seen on biopsy but are not necessary to make the diagnosis. In fact, the degree of eosinophilia has been shown to not correlate with disease severity. Therefore, laboratory results are not helpful in following disease activity [1, 11, 12]. Some patients have displayed normalizing laboratory results with persistent clinical evidence of EF [5, 11].

The cornerstone of treatment for eosinophilic fasciitis has largely been corticosteroid therapy. Multiple studies have shown their effectiveness in the majority of cases, although steroid-refractory cases and relapses have been documented in medical literature. NSAIDs, aspirin, phototherapy, cyclosporine, hydroxychloroquine, cimetidine, azathioprine, and d-penicillamine have all been used for the treatment of eosinophilic fasciitis [2, 4, 5, 13].

## 2. Case Report

A 48-year-old right-hand dominant Caucasian female reported to the emergency department with complaints of left hand redness, swelling, warmth, pain and tenderness, on the left dorsal surface of the hand located over 2nd and 3rd MCP joint. She had reported having “poked/stabbed” and subsequently punctured the skin of her left hand with kitchen shears 4 days prior. The symptoms began immediately after the incident 4 days ago, but this afternoon there was increased pain and swelling with full extension of second digit on the left hand.

Initial patient consultations were made by the orthopedic department. The patient had no cough or respiratory distress, although the patient did have a history of asthma. Review of the endocrine system revealed no history of thyroid disease. There was a normal range of motion in all four extremities, and distal pulses were “normal.” Evaluation of the wound by medical professionals revealed a laceration located at the left dorsal radial aspect of 2nd digit with erythema and mild swelling located over 2nd and 3rd MCP joints. No discharge or fluctuance was noted at the wound site. Tenderness was elicited over the 2nd digit on the left upon palpation, and pain with full extension of finger was experienced. Overall, the patient was noted to have full range of motion and was neurovascularly intact at the left hand.

Three plain film radiographic views of the left hand were taken, which revealed “no evidence of traumatic osseous pathology or bony abnormality…impressions: normal left hand.” Laboratory blood draws revealed slightly elevated basophils (3%, compared to the normal of. 0.0–1.0% reference range), as well as slightly elevated basophil antibodies (0.3 × 10^3^/cumm as compared to the reference range of 0.0–0.2 × 10^3^/cumm). Otherwise, all the other values analyzed in the complete blood count with differential were essentially unremarkable. Two blood cultures were taken at this time, both revealing no growth.

At the emergency department she was treated with one dose of IV antibiotics and then was discharged with a prescription for Cephalexin. She took the Cephalexin for the full ten-day course. The patient was admitted to self-medicating with Ibuprofen and Acetaminophen as needed, for the pain and inflammation. She also expressed the use of Diphenhydramine. The patient noted improvement in the swelling and pain.

She reported back to the emergency department a couple weeks later, after she got hit in the dorsum of the left hand by her dog's paw. She subsequently began to notice increased erythema and pain at the left hand. She now noticed numbness on the radial aspect of the left index finger. Evaluation of the site revealed mild edema on the dorsum of the second web space between second and third MCP joints. There was mild erythematic in this area, with tracking proximally up the dorsum of the forearm.

Review of systems was unremarkable. The patient denied constitutional symptoms, and while in the emergency department the vitals were stable and the patient was afebrile. However, the patient commented she had taken her temperature at home 2 days prior and it was 103.0 degrees Fahrenheit. Complete blood count with differential revealed slight elevation in basophils, at 1.8%. The CBC with differential was otherwise unremarkable. Diagnostic X-ray imaging of the left hand revealed findings suggestive of cellulitis with lymphangitis. The orthopedic department upon review of the case suggested the patient be admitted and given IV antibiotics, warm compresses to the left hand, and pain control as needed. Against medical advice, the patient went home on oral Clindamycin. The patient continued to self-medicate with Ibuprofen and Acetominophen.

The patient reported back to the emergency department almost 3 weeks later with the chief complaint of swelling and pain on the dorsum of the left index finger MCP joint, as well as erythema which tracked up to the midforearm. A complete blood count with differential revealed a slight elevation in basophils, at 1.3%. Findings suggested left hand pain secondary to cellulitis with ascending lymphangitis. The patient had failed outpatient therapy, and the orthopedic department suggested inpatient admission, IV antibiotics, application of warm compresses, and close monitoring. The patient stated that due to financial reasons she would refuse admission, and she left with a prescription for oral antibiotics (Cephalexin, and Sulfamethoxazole and Trimethoprim) and NSAIDs for the pain and inflammation.

Two months later the patient reported back to the emergency department with complaints of left hand swelling and an increase in pain and redness. The patient states that two days ago the swelling and the redness began and progressively worsened until presentation to the emergency department. A fever of 101.0 degrees Fahrenheit was measured at home and treated with Acetominophen. Two separate blood cultures were taken at this time, and they both revealed no growth. The complete blood count with differential revealed no abnormalities. CT scan with IV contrasts was undertaken for evaluation of the left hand for a possible abscess or foreign body. This revealed no radiopaque foreign body, although there was indeed soft tissue swelling of the dorsum of the hand extending into the index finger. No discrete abscess collection was seen. There were no osseous changes seen on CT to suggest osteomyelitis. Radiographs of the left hand revealed “normal” findings. Imaging of the patients suggested cellulitis according to radiology reports.

Discussions between infectious disease physicians and orthopedic surgeons led to the presumption of chronic left hand cellulitis with ascending lymphangitis. The patient was admitted to the hospital on IV Vancomycin and Ancef. Orthopedic surgeons evaluated the patient for possible surgery but she was deemed to be stable with no need for surgical intervention at the time. The patient remained stable through her entire hospital stay, and after 2 days of inpatient therapy, she was discharged in stable condition with instructions for outpatient follow-up and oral antibiotic of Cephalexin.

Eight months later the patient reported to the emergency department with complaints of a red swollen left hand for about 3 days. The patient complained of left hand pain that began while at rest. The symptoms had been constant, described as an 8/10 stabbing pain to the left hand region. Findings in the affected area included decreased range of motion. The patient was given morphine sulfate while in the emergency department and the pain subsided. The patient left the hospital with instructions to follow up with her primary care physician in the outpatient setting. The patient failed to do this, and she reported back to the emergency department with the same symptoms, just three days later. At this point the white blood cell count was elevated, at 14.5 × 10^3^/cumm (4.3–10.0 × 10^3^/cumm reference range). The pain was now 10/10, described as aching and throbbing in nature. Radiographic imaging of the left hand revealed swelling, but otherwise it was a “normal hand.” The patient had been experiencing anxiety and depression, which she attributed to the constant bouts of pain. The patient had also been experiencing debility secondary to the decrease functioning and use of her left upper extremity. At this point in time she screamed out in a fit of distress

“just take my arm off please …. I cannot deal with this anymore.”

Due to the nature of the patient's condition, orthopedic surgeons decided to operate. Based on the intensity of the swelling, erythema, and pain, concern was present for a possible necrotizing fasciitis. The preoperative diagnosis was left hand and forearm possible acute necrotizing fasciitis. During the procedure a left hand and forearm incision was made, and irrigation and debridement were accomplished. No purulent material was identified. There was some fibrinous exudate around the extensor mechanism overlying the dorsum of the hand. The fibrous exudate was debrided where necessary and sent for tissue culture. Myofascial biopsy of left hand and left forearm were also taken at this time. The muscle and fascia did not demonstrate any areas of necrosis to the naked eye. The biopsy was discussed with pathologists intraoperatively while the procedure was underway, and the conclusion was made that the forearm myofascial biopsy did not demonstrate any evidence of necrosis, but the left hand demonstrated early acute suppurative myositis and fasciitis, consistent with a necrotizing fasciitis. It was decided to treat without aggressive debridement and amputation. Gram stains of the left hand and forearm revealed no organisms seen, although there were 3+ PMNs noted. There was not growth on acid fast bacilli smears, fungus cultures, aerobic and anaerobic cultures, or blood cultures. CBC with differential revealed a mild leukocytosis at 13.4, without eosinophilia. Rheumatoid factor levels were normal, and an ANA screen was negative.

During this same hospital stay, the patient was once again taken back for irrigation and debridement. Samples for culture also came back negative for growth. She underwent a third surgical procedure for treatment of left middle finger tenosynovitis. A tenovaginotomy and A1 pulley release of left middle finger was accomplished, along with irrigation and debridement. The patient was kept in the hospital until she was in stable condition, and then was discharged with instructions to follow up with orthopedic surgeon in the outpatient setting.

Less than two weeks after discharge the patient once again reported back to the hospital with similar complaints of left arm and hand pain. The severity was 10/10 and described as “burning” in nature. The left hand displayed significant erythema, induration, and fluctuance over the dorsal aspect. At this time a CBC with differential was ordered and the findings revealed a leukocytosis of 13.0 × 10^3^/cumm, neutrophils at 92.8% (42.2–75.2% reference range), eosinophils within normal range, elevated CRP at 16.4 mg/dL (0.0–1.0 mg/dL reference range), CPK (creatine kinase) within normal range, and RBC sedimentation rate at 54 mm/hr (0–20 mm/hr reference range). The decision to take the patient pack to the operating room was made for presumptive left hand cellulitis, presumed left dorsal hand abscess, and presumed left ring finger purulent tenosynovitis. Incision, irrigation, and drainage were preformed, and biopsies were taken intraoperatively of the left hand and forearm. No purulence or abnormal-looking tissue was noted during the procedure. All cultures taken intraoperatively came back to reveal no growth once again. Pathology revealed startling new findings at this time. An “eosinophil-rich superficial and deep mixed inflammatory cell infiltrate” was noted. This pathological specimen was compared to that previously taken, which revealed early acute suppurative myositis and fasciitis consistent with a necrotizing fasciitis. In the prior specimen, there was a mixed cell fasciitis that included eosinophils, which suggests the possibility of eosinophilic fasciitis, but not the eosinophil-rich superficial and deep cutaneous infiltrate as currently noted. There was a panniculitis and fasciitis overlying focal epidermal spongiosis, suggesting a hypersensitivity reaction, as expected following an arthropod assault, allergic contact dermatitis, or a drug eruption. Though it was presumed to be early necrotizing fasciitis, the patient's unnatural history and her inability to improve was not consistent with the patient's presentation. Pathologists in discussion with orthopedic, infectious disease, and rheumatology physicians made the diagnosis of eosinophilic fasciitis. The patient was subsequently placed on steroids and her symptoms improved.

Years before the symptoms of eosinophilic fasciitis occurred, the patient was diagnosed with asthma. She had experienced multiple hospital visits and exacerbations of her asthma, before, during, and after the diagnosis of eosinophilic fasciitis has been made.

## 3. Discussion

Through the review of the literature, there is only one other case by Yamanishi where recurrent asthma has been seen to be associated with eosinophilic fasciitis [8]. To the knowledge of the authors of this paper, this patient represents the second recorded incident. There was a prior case report by Gabrielli [14] that displayed the onset of eosinophilic fasciitis with the simultaneous development of asthma. Unlike the Gabrielli patient, this patient had a long history of asthma prior to the emergence of EF.

The Gabrielli case report demonstrated mast-cell-rich (not eosinophil) tissue biopsies. The Yamanishi patient linking recurrent asthma with eosinophil-fasciitis demonstrated eosinophilic rich tissue biopsies. The case described by the authors of this paper, demonstrated an initial biopsy of mixed cell fasciitis including eosinophils, compared to the eosinophil-rich sample taken at a later date. This could be a unique aspect to the pathology of the disease not previously discovered. Similar scenarios were not noted in a review of the literature. A change in the pathological findings as shown in this case from non-eosinophil-rich sample to one rich in eosinophils is unique in a patient actively suffering from EF.

The authors of this paper propose that an allergic reaction (at the patient's puncture site) occurred, which initially caused the left hand symptoms that led to the patient's first presentation to the hospital. This is a unique causative agent, not found in the review of the literature. This could have triggered the eosinophilic fasciitis that would later develop or did indeed develop at that time. The rapid/immediate onset of symptoms of left hand pain, swelling, and erythema, along with negative cultures, points to the possibility of an allergic reaction causing the initial hand symptoms. Although trauma has been linked as a possible cause of eosinophilic fasciitis, the patient's ectopic history leads credence to the fact that there could in fact have been an allergic reaction at the site. The patient has a long history of asthma and is allergic to penicillin. A slight basophilia at the initial presentation could be have had ectopic origins. The patient had an increase in basophils; although slight, it was above the patient's baseline. One must keep in mind that the patient presented 4 days after the inciting incident. In which time, the basophil count could have already peaked and been on the decline by the time the patient reported to the emergency department. The patient at the time was taking anti-inflammatory medications, a steroid inhaler for her asthma, and Diphenhydramine, which may have blunted the allergic reaction and basophilia. The proposed allergic reaction, although relatively minor in nature, could have been all that was needed to trigger the EF.

One must keep in mind that eosinophilic fasciitis has also been associated with Hashimoto's thyroiditis, an autoimmune disease. The triggering of EF in this case by a supposed allergic reaction, and the link to asthma as shown in this case and in others, also links the disease to an underlying immunological origin. All these links to the body's immune system could represent an underlying dysregulation of the immune system, leading to the initiation or synergistic perpetuation of EF.

The patient made repeated visits to the emergency department with complaints of hand pain, swelling, and so forth. The patient was repeatedly given antibiotic treatment and pain medications for supposed cellulitis. She was also taking Ibuprofen, NSAIDs, and Acetominophen at home for the inflammation and pain. In retrospect, it was probably the anti-inflammatory medications, along with the systemic effects of the albuterol steroid inhaler she was taking for asthma, which quelled the symptoms of the eosinophilic fasciitis. The multiple cultures came back negative for any sort of growth, discrediting efficacy of antibiotics in the relief of the patient's symptoms. The patient's symptoms could have just subsided and reemerged on their own, which has been seen in previous cases of EF. The condition has been shown to have symptoms that are persistent or recur during the course of the disease.

## 4. Conclusion

The presentation of this case was unique and unusual. The patient seemed to improve with the antibiotic therapy, lending support to the diagnosis of cellulitis or a necrotizing process. Pathology reports pointed to a disease process other than eosinophilic fasciitis. These all lead to a delay in the diagnosis of EF. The extreme rarity of the disease, the large constellation of signs and symptoms, and the lack of knowledge about eosinophilic fasciitis make this disease difficult to recognize and treat.

The orthopedic surgeon must be able to recognize the causative agents, symptoms, and pathology of this disease process in order to determine the need for surgical care, versus that of pharmacological therapy. Therapy such as corticosteroids and NSAIDs has proven efficacy in treating this condition. The well-informed orthopedic surgeon needs to be able to recognize the unique pathology of eosinophilic fasciitis as described in this case and apply it in making the correct diagnosis. Proper diagnosis and treatment by the orthopedic surgeon can circumvent debility secondary to pain and the musculoskeletal sequela of this disease process.
